# How ceramides affect the development of colon cancer: from normal colon to carcinoma

**DOI:** 10.1007/s00424-024-02960-x

**Published:** 2024-04-18

**Authors:** Nadine Merz, Jennifer Christina Hartel, Sabine Grösch

**Affiliations:** 1 Goethe-University Frankfurt, Institute of Clinical Pharmacology, Theodor Stern Kai 7, 60590 Frankfurt, Germany; 2 Fraunhofer Institute for Translational Medicine and Pharmacology ITMP, Theodor-Stern-Kai 7, 60596 Frankfurt Am Main, Germany

**Keywords:** Sphingolipid, Colon cancer, Ceramide synthase, Hypoxia, Crypt, Stem cell

## Abstract

The integrity of the colon and the development of colon cancer depend on the sphingolipid balance in colon epithelial cells. In this review, we summarize the current knowledge on how ceramides and their complex derivatives influence normal colon development and colon cancer development. Ceramides, glucosylceramides and sphingomyelin are essential membrane components and, due to their biophysical properties, can influence the activation of membrane proteins, affecting protein–protein interactions and downstream signalling pathways. Here, we review the cellular mechanisms known to be affected by ceramides and their effects on colon development. We also describe which ceramides are deregulated during colorectal carcinogenesis, the molecular mechanisms involved in ceramide deregulation and how this affects carcinogenesis. Finally, we review new methods that are now state of the art for studying lipid-protein interactions in the physiological environment.

## Introduction

Colon cancer is the third most common cancer death worldwide. The outcome of colon cancer patients is strongly dependent on the stage at which the tumour is diagnosed. While patients with UICCI (Union for International Cancer Control I) or UICC II tumours have a 5-year survival probability of about 90%, it drops to 60–70% in UICC stage III and worsens to only 10–20% at stage IV [31, 104]. Various molecular pathways have been identified to be involved in the development of colon cancer. The best known is the genetic model of colorectal tumourigenesis from Fearon and Vogelstein identifying tumour driver mutations in the adenomatous polyposis coli (APC), K-ras or p53 gene [28]. Today, we know that besides these genetic mutations, several other genetic markers are also important to characterize colon tumours properly and to improve the prediction of prognosis and treatment options [10].

**Fig. 1:**
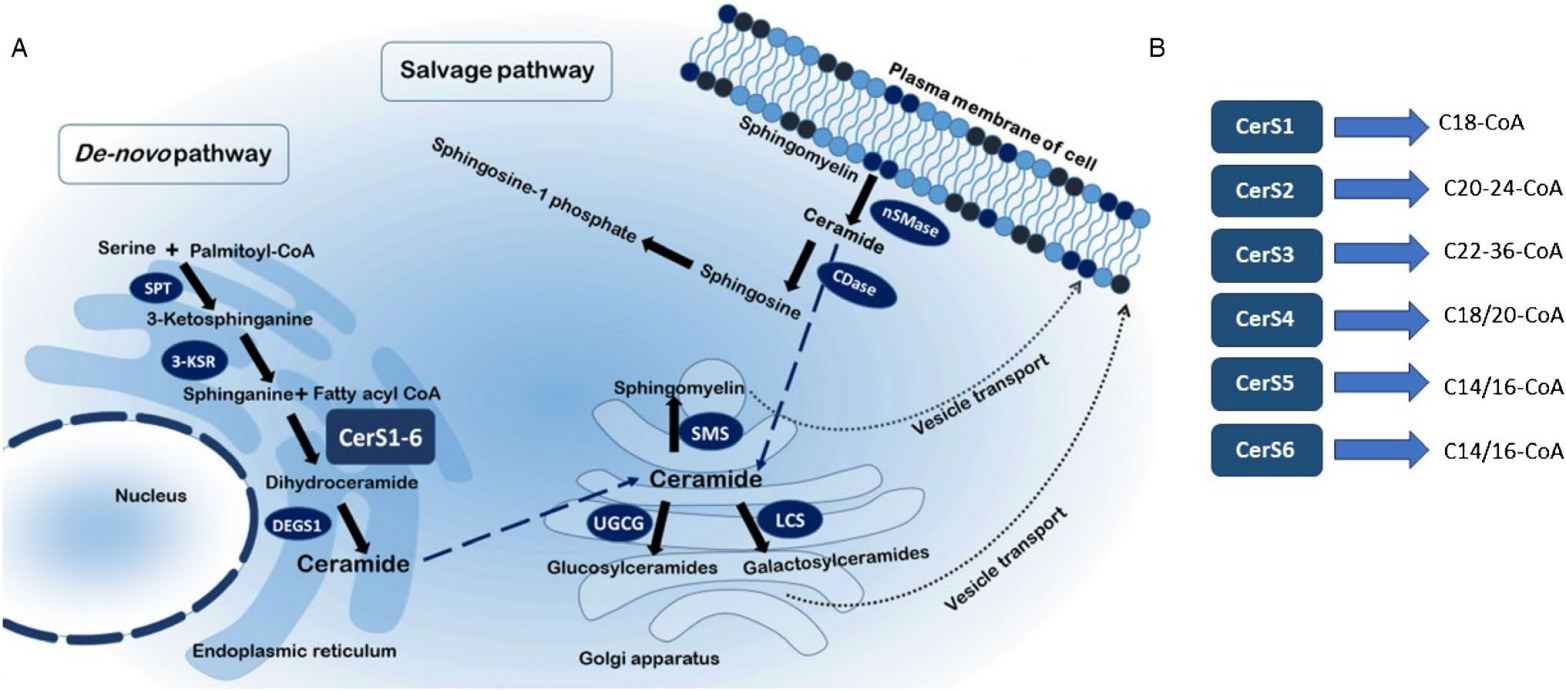
Pictogram of the sphingolipid pathway. **A** De novo synthesis and salvage pathway of the sphingolipid pathway (see also description in the text). **B** The different mammalian CerS and their preferred acyl-CoA substrates for the generation of dihydro-ceramides or ceramides. SPT, serine palmitoyl transferase; 3-KSR, 3-ketosphinganine reductase; CerS, ceramide synthase; DEGS, dihydroceramide desaturase; SMS, sphingomyelin synthase; UGCG, UDP-glucose ceramide glucosyltransferase; LCS, lactosylceramide synthase; nSMase, neutral sphingomyelinase; CDase, ceramidase

In the last few years, several groups investigated the lipid profile of cancer cells [86, 92]. Lipids are not only energy stores but are also essential structural components of membranes; therefore, the high proliferation rate of cancer cells requires large amounts of lipids as building blocks for biological membranes [86]. Furthermore, it has been shown that the lipid composition changes during cancer development, indicating that lipids are not only inactive structures of membranes but are also important signalling molecules influenced by or involved in cancer development and could be used as biomarkers for staging cancer [42, 55]. Sphingolipids are one of these lipids which share both functions: important membrane components and signalling molecules. Sphingolipids can be generated in mammalian cells via de novo synthesis and are metabolized in the *salvage pathway*. The de novo synthesis starts in the endoplasmic reticulum with the condensation of L-serine and palmitoyl-CoA by serine palmitoyl transferase (SPT). The intermediate 3-ketosphinganine is directly reduced to sphinganine by the activity of 3-ketosphinganine reductase (3-KSR). Ceramide synthases (CerS) add fatty acyl-CoAs of different chain length to the sphingosine backbone to generate dihydroceramide (dhCer). The enzymes, dihydroceramide desaturases 1 and 2 (DEGS 1/2), insert a 4–5 trans-double bond and convert, thereby, dhCer into ceramide. After de novo generation, ceramides are transported to the Golgi apparatus, where they are metabolized to complex sphingolipids. Here, the ceramide is assembled to form glucosylceramide (GluCer) via the UDP-glucose ceramide glucosyltransferase (UGCG), lactosylceramide (LacCer) by the activity of the lactosylceramide synthase (LCS) and sphingomyelin (SM) by the activity of sphingomyelin synthases or ceramide-1-phosphate (C1P) by ceramide kinase. The complex sphingolipids can be transported via vesicles from the Golgi apparatus to the plasma membrane and other cell organelles. Degradation of sphingolipids is mediated by the salvage pathway in lysosomes or at the plasma membrane by the action of sphingomyelinases (SMase) and ceramidases (CDase) (Fig. 1A and B).

Ceramides, which are the central molecules of complex sphingolipids, act in a chain length–specific manner [34]. They are essential for maintaining cellular membrane functions, for example, as shelters from the environment or in intracellular compartmentalization. Furthermore, they are important constituents of special membrane microdomains, so-called lipid rafts, that are enriched in cholesterol and sphingolipids and function as signalling hubs by conglomeration of different proteins to multi-protein complexes necessary for signal transduction [42, 67]. About one-third of all proteins are predicted to be embedded in biological membranes [94]. The activity or functionality of these proteins are influenced by surrounding lipids, and many of these proteins are growth factor receptors or important for cell metabolism. Ceramides of specific chain length can influence these membrane proteins. Therefore, we want to highlight the role of ceramides in colon physiology and the development of colon cancer.

## Ceramides are important for colon homeostasis

**Fig. 2:**
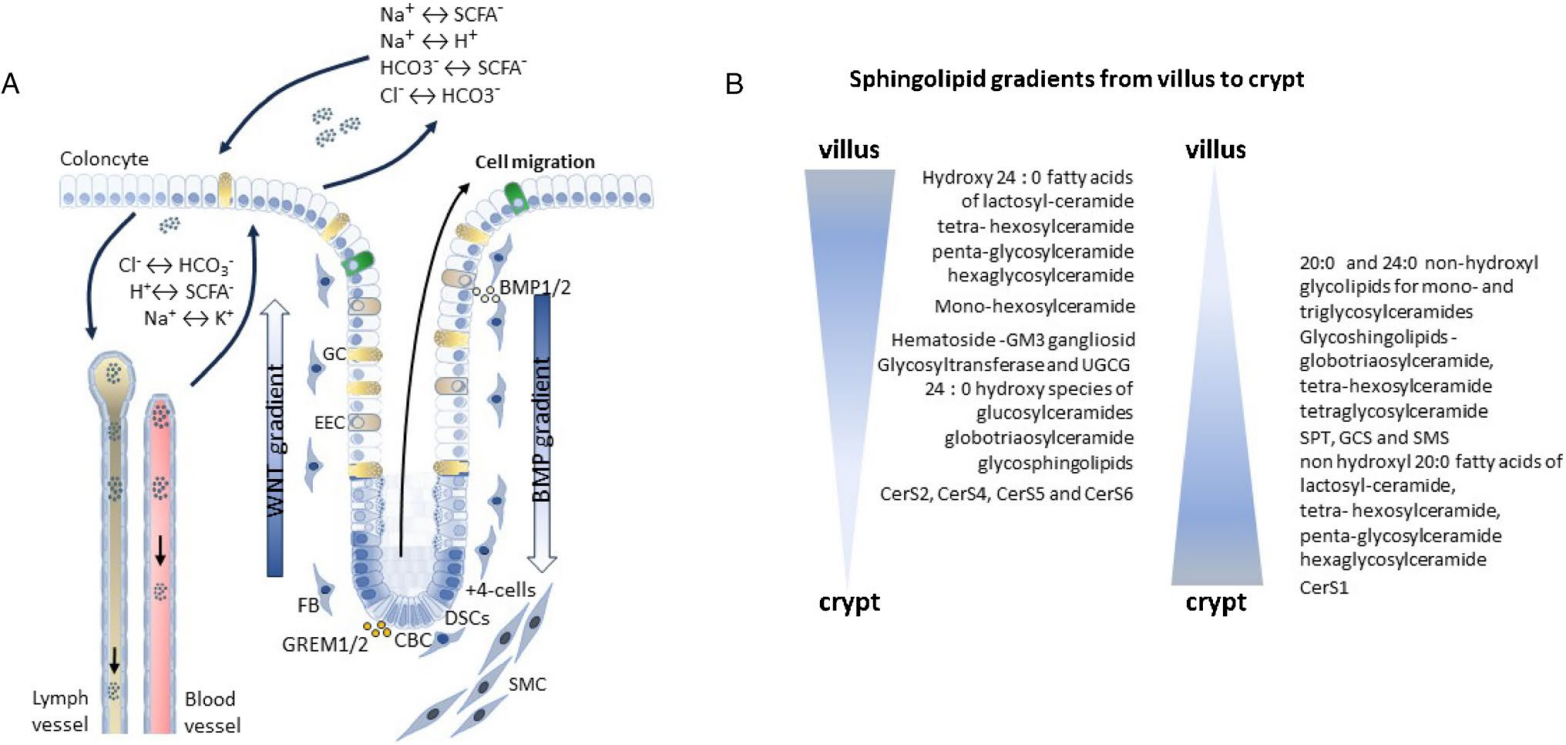
Organization of colon crypts, physiological function of the colon and sphingolipid gradients in the intestine. **A** The stem cell niche is built up by crypt-base columnar cells (CBCs), deep secretory cells (DSCs), + 4 cells, fibroblasts (FBs) of different subtypes and smooth muscle cells (SMCs). FB and SMC secrete at the crypt bottom gremlin 1 and 2 (GREM1/2) and at the top of the crypt the bone morphogenic protein 1 and 2 (BMP1/2). Water, ions and short-chain fatty acids (SCFAs) such as acetate, propionate and butyrate were generated by diverse population of bacteria, taken up by the colonocytes and are further transported to the lymph- and blood vessels (for further description, see text). **B** Differentiated villus cells and immature crypt cells differ strongly in the sphingolipid composition in the intestine, data from [6, 7, 11, 12]

The colon architecture is characterized by epithelial invaginations, so-called crypts. The main function of the colon is the resorption of water and ions (Fig. 2A). The crypts are lined by a unicellular epithelial layer that renews itself every 4 to 5 days. At the bottom of the crypts, the colon stem cells (also known as crypt-base columnar cells, CBCs) are located from which all colon epithelial cell descent. They reside in a special niche (stem cell niche) that produces signals to control and support stem cell behaviour [5, 108]. The niche is built up by deep secretory cells (DSCs), + 4 cells, fibroblasts of different subtypes and smooth muscle cells [93] (Fig. 2A). Communication in the stem cell niche between epithelium and its surrounding takes place bidirectionally and is important for the maintenance of CBCs but also for the different stromal cells [60]. Myofibroblasts and smooth muscle cells secrete fibronectin and TAGLN (transgelin) but also the bone morphogenic protein (BMP) inhibitors gremlins 1 and 2 (GREM1/GREM2) that influence epithelial cells at the bottom region of the colon crypt [54]. Instead, in the upper region of the crypt, proliferation-inhibiting and differentiation-promoting genes are expressed like SAMD7 and BMP1 BMP2, leading to the differentiation of epithelial cells into enteroendocrine, secretory (goblet cells) or absorptive cells (enterocytes) [32]. Therefore, along the intestinal crypt, variable cellular signalling pathways are activated in the epithelial cells that are important to maintain the development of the different cellular subtypes which require a perfect balance sphingolipid distribution that is depicted in Fig. 2B. As mentioned above sphingolipids are building blocks of the membranes and are involved in the formation of lipid rafts [51] that are important for the functionality of the membrane imbedded or associated proteins [18, 75]. In the colon epithelial cells, sphingolipids are essential. This has been proven by the generation of intestinal epithelial-specific serine palmitoyltransferase (SPT)-knockout (ko) mice using Sptlc2 Villin (Vil)-Cre knockout (ko) mice. SPT is the first and rate-limiting enzyme of the sphingolipid de novo synthesis. The enzyme consists of three subunits—SPTLC1, SPTLC2 and SPTLC3—where SPTLC1 and SPTLC2 are ubiquitously expressed and SPTLC3 is restricted to specific tissues [61]. Data from the Human Protein Atlas show a moderate expression of each subunit in colon tissue [70, 96]. SPTLC1 is essential for the activity of the enzyme. However, SPTLC1 can form a complex with SPTLC2 and/or SPTLC3, which results in the formation of long-chain bases with varying aliphatic chain lengths [44, 61]. Sptlc2-VilCre ko mice are not viable, and also tamoxifen-inducible Sptlc2-Vil-CreER ko mice die within few days [59]. In these mice, the crypt structure was disorganized (crypts of Lieberkühn and villi structures are only partly developed, microvilli are shorter and rudimentary), and barrier function strongly impaired few days after ablation of Sptlc2 by tamoxifen. This goes along with a reduction of goblet cells, loss of mucin 2 and intravasation of bacteria into the colon epithelium [59, 73]. These data strongly imply that SPT and therefore ceramides are essential lipids to maintain colon homeostasis. In this context, Li et al. have shown that SPT is an essential enzyme of small intestinal stem cells [58]. They demonstrate that SPTLC2 is highly expressed in the crypt than in the villi of the small intestine. Using specific inhibitors of glucosylceramide synthase, sphingomyelin synthase or ceramide synthases in intestinal organoids indicate that the sphingolipid de novo synthesis is important for organoid survival. Mechanistically, it has been shown that the fatty acid–binding protein 1 (FABP1) and the carnitine palmitoyltransferase-1$\alpha$ (CPT1$\alpha$) which facilitate lipid uptake into cells or mitochondria, respectively, are most affected by a depletion of ceramide de novo synthesis in organoids [58]. Together, these data indicate that the sphingolipid de novo synthesis is indispensable for epithelial cells of the intestine and important for cell survival.

SPT generates 3-ketophinganine, which is a highly chemically reactive intermediate that is directly reduced to sphinganine by the 3-ketophinganine reductase. This enzyme is localized close to the SPT in the endoplasmic reticulum (ER) and, until now, is likely the sole enzyme responsible for the formation of sphinganine from 3-ketosphinganine [35]. Not much is known about this enzyme, but as it is the only known enzyme that catalyses this reaction, it can be assumed that it is as essential for the sphingolipid de novo synthesis as the SPT. But further studies must prove this hypothesis.

Sphinganine is acetylated at the primary amine group by CerS with an acyl-CoA of different chain length to form dihydro-ceramides (dh-Cer) (Fig. 1A). In addition to sphinganine from de novo synthesis, sphingosine from the salvage pathway is also used as a substrate by CerS, leading to the production of ceramides. Six different CerS are known in mammals, which differ in their tissue expression and substrate specificity with respect to different acyl chain lengths (Fig. 1B). However, some CerS have overlapping substrate specificities and can therefore compensate for each other. From CerS knockout (ko) mice, we know that depletion of one CerS is in part compensated by up-/downregulation of other CerS, resulting in a shift of the ceramide equilibrium, which is important for cell fate [34, 37]. A very comprehensive review from Park et al. depicts the complex interplay between CerS and how they influence each other [76]. However, each CerS ko mouse has its own specific phenotype, indicating that in certain tissues the loss of one CerS could not be compensated by others [23, 24, 33, 46, 81–83, 116]. Sphingolipids and cholesterol are primarily responsible for the conversion of loosely packed membranes into rigid one, which directly impacts the location of membrane associated proteins into lipid rafts. The lipid composition of membranes is therefore tightly regulated and plays a decisive role in the function of membranes [43]. Several groups have shown that the glycosphingolipid level in colon epithelial cells differ strongly along the crypt-villus axis (in their amount of glycosylation as well as in their chain length) and proposed that this is associated with the various cellular requirements of proliferating and differentiating cells (Fig. 2B) [6, 7, 11, 12]. The expression level of the enzyme that is responsible for the first glycosylation step of ceramides, UDP-glucose ceramide glucosyltransferase (UGCG), is highly expressed in the villi in comparison to the crypt [54]. Intestinal stem cells are highly dependent on factors that are produced by surrounding cells (like R-spondin, EGF, BMP, signalling inhibitor noggin, WNT) which binds to receptors at the stem cell plasma membrane [106]. Many of these receptors are located in lipid rafts and therefore dependent on the composition of membrane lipids [2, 74, 100, 101]. WNT is one of the main factors regulating intestinal stem cell maintenance and proliferation by binding to the Frizzled-LRP5-LRP6 receptor complex at the plasma membrane thereby activating the $\beta$-catenin transcription pathway [32]. Activation of the Frizzled-LRP5-LRP6 receptor complex leads to its translocation into lipid rafts and its interaction with the coated vesicle-associated kinase of 104 KDa (CVAK104) mediates its internalization and degradation [95]. CVAK104 (also known as SCY1-like protein pseudo-kinase 2; SCYL2) and the serine-threonine kinase 38 (STK38), which is involved in the phosphorylation of glycogen synthase kinase 3β (GSK3$\beta$) leading to the degradation of $\beta$-catenin [49], “colocalize” with CerS2 in prostate cancer cells [114]. Overexpression of wt CerS2 leads to the degradation of $\beta$-catenin, whereas a mutated CerS2 protein, which is mutated at a putative phosphorylation site of CerS2, enhances $\beta$-catenin expression in prostate cancer cells and enhances cancer growth and metastasis [114]. How CerS2 affects CVAK104 or STK38 activity is not clear. Because the Frizzled-LRP5-LRP6 receptor is located at the plasma membrane (PM) and CerS2 is located at the ER, physical contact sites between the ER and the PM might favour this interaction. An alternative hypothesis might be that mutation of the phosphorylation site in CerS2 has an impact on CerS2-activity, because CerS2 enzyme activity is regulated by phosphorylation [87]. But this has not been shown by the authors. It is unclear whether direct contact between CerS2 and CVAK104/STK38 or an increase in very-long-chain ceramides influences the $\beta$-catenin signalling pathway. In this context, we want to refer to a very good review from Alonso and Goni summarizing the physical properties of ceramides on membranes, showing that depending on the chain length of ceramides the behaviour of liquid-ordered domains and gel domains differs in the membrane [1]. It has been shown that CerS2 is twofold highly expressed at the top of colon crypts in comparison to crypt bottom epithelial cells (Fig. 2B), but it must be checked if the effect of CerS2 on $\beta$-catenin signalling observed in prostate cancer cells also plays a role in colon crypts and if phosphorylation differences in CerS2 are involved. However, untreated CerS2 knockout mice weighed 20–30% less than wt mice [82] and had a disturbed colon barrier function (see below). Weight loss in CerS2-ko mice might be related to an altered nutrition absorption in the small intestine in these mice, as the resorption takes place at the plasma membrane by the activity of various transporters and receptors. The insulin receptor in the intestinal epithelium is important for intestinal gene expression and glucose uptake [99]. Park et al. could demonstrate that in CerS2-ko mice phosphorylation and signalling of the insulin receptor are impaired which correlate with its inability to translocate into detergent-resistant membranes [77]. Therefore, an improper function of the insulin receptor in the intestine of CerS2-ko mice might be jointly responsible for the observed weight loss in these mice. In contrast, CerS4-6 ko mice show no phenotype regarding the functionality or morphology of the colon, indicating that the loss of these CerS could be compensated by others to maintain colon homeostasis. Nevertheless, after treating CerS-ko mice with dextran sodium sulphate (DSS) and azoxymethane (AOM) to induce colitis or colon cancer CerS2-6 ko mice react differently (see below).

In summary, the sphingolipid content in the intestine is tightly regulated between the villi and the crypt. Changes in the sphingolipid equilibrium influence the physicochemical properties of cell membranes which have a direct impact on imbedded membrane proteins that are required for differentiation of stem cells into enteroendocrine, secretory or absorptive cells.

## Ceramides influence early- to late-stage colon cancer

Colon cancer either develops spontaneously or is driven by genetic predisposition like hereditary non-polyposis colorectal cancer (HNPCC), familial adenomatous polyposis (FAP) or the MUTYH-associated polyposis (MAP). Furthermore, different diseases like Peutz-Jeghers syndrome (PJS), cystic fibrosis (CF), inflammatory bowel disease (IBD) or type 2 diabetes increase the risk to develop colon cancer. The risk factors for spontaneously arising colon cancer are overweight (obesity); red and processed meats; fried, broiled, or grilled food; smoking; alcohol; and age. So, some risk factors could be influenced by lifestyle changes, but age and genetic predispositions are risk factors that can only be minimized by regular check-ups. The most common colon cancer is the adenocarcinoma (about 95%) that descends from secretory epithelial cells. One of the first tumour driver mutations in colon cancer occurs in the adenomatous polyposis coli (APC) gene. About 80–90% of all colon tumours show mutations in this gene [113]. Mutations in K-ras and p53 are further tumour driver mutations leading to the manifestation of colon tumours [28]. In organoids, derived from mice small intestine, loss of APC leads to a dramatic upregulation of ceramides and hexyl-ceramides, whereas the level of some sphingomyelin decreased [48]. How these changes in the sphingolipid profile are related to APC mutations has not been investigated. Another group showed that especially the alkaline sphingomyelinase (alkSMase) is decreased in colon adenocarcinoma [22] and that loss of alkSMase is associated with colon tumorigenesis [16, 40]. However, reduction of alkSMase is not related to APC gene mutation [41]. In addition, sphingomyelin synthase 2 ko mice are less susceptible to AOM/DSS-induced colon cancer [72]. Therefore, the data from mouse organoids seem to be contradictory to the previously published data. But the lipidomic screening of mice organoids show only data for SM (d18:1/14:0) and SM (d18:1/16:0) and not for other very-long-chain SM. From human colon cancer lipidomic screening, we know that we must look in more detail at the lipidomic data, as not all SM or Cer decrease or increase in colon tumours, but changes occur in a chain length–dependent manner (see the next chapter). Therefore, changes in the sphingolipid metabolism are likely to impact colon cancer development, but it must be carefully looked at the specific lipid species also within one lipid group. The lipidomic screening of sphingolipids in colon cancer tissue and in plasma from colon cancer patients indicates that sphingolipids, especially ceramides, are deregulated in colon cancer patients [25, 42, 56, 64, 89]. In colon tumours, a decrease in long-chain ceramides (C18:0- and C20:0-Cer) has been detected in comparison to control colon tissue by different groups [25, 42]. Together with changes in other lipids (SM and triacylglycerol (TG) species), these lipids could be used to differentiate colon tumour tissue from normal colon tissue (see below) [25]. They are predicted to be additional biomarkers for patients with colon cancer and significant prognostic markers for patient outcome. However, only few of these publications investigated the molecular mechanisms that lead to changes in sphingolipids or how sphingolipids influence colon cancer. Here, we want to give an overview about the sphingolipids, especially ceramides, that are deregulated in colon cancer and how this might affect colon cancer development.

## CerS4 in colon cancer development

**Fig. 3:**
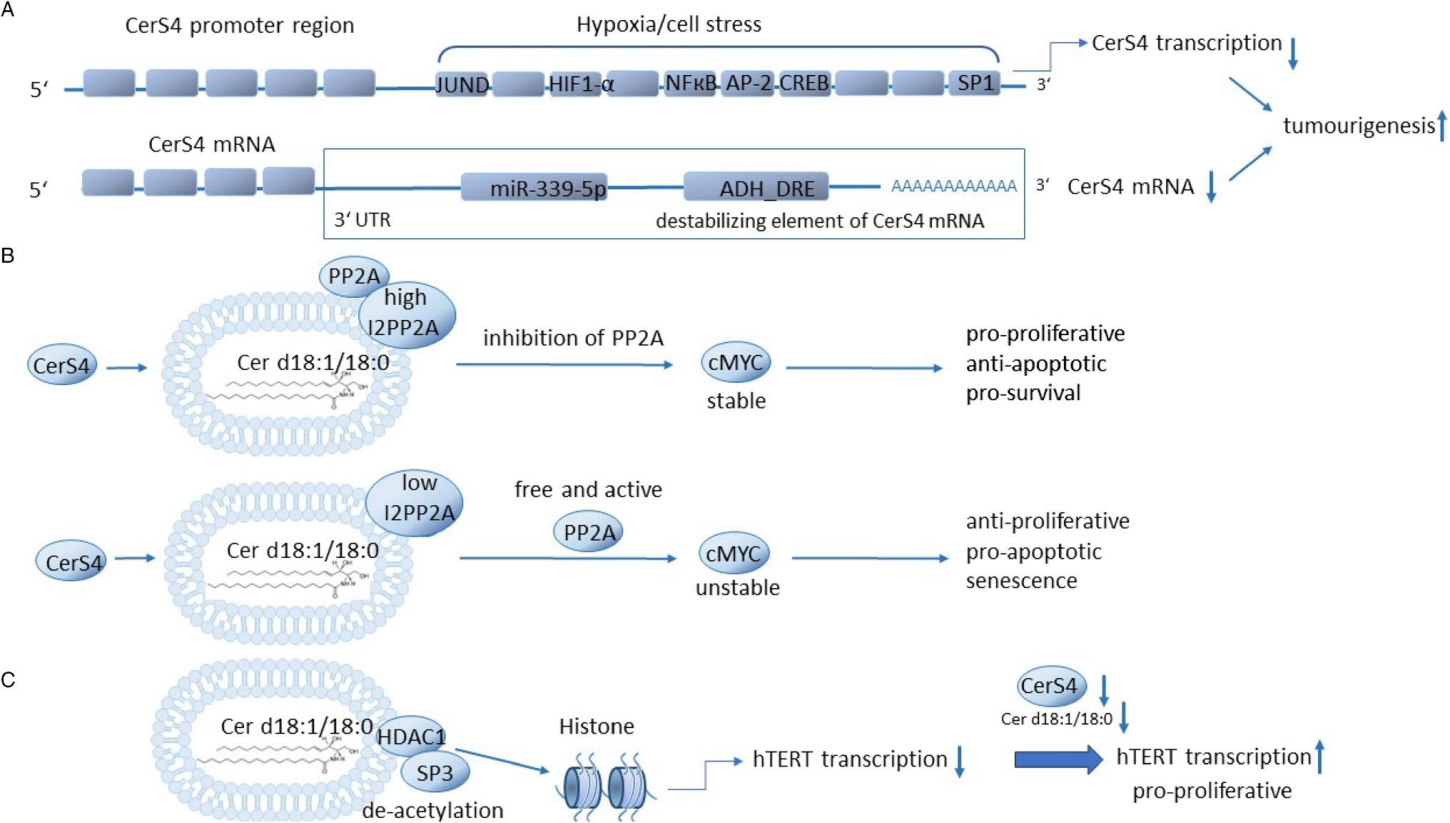
CerS4 in colon cancer development. **A** Transcriptional and posttranscriptional regulatory elements that might inhibit CerS4 transcription or lead to the instability of CerS4 mRNA (for description, see also text). **B** Interaction of C18-Cer with inhibitor 2 of protein phosphatase 2A (I2PP2A) can have pro-proliferative and anti-proliferative effects depending on the expression level of I2PP2A. **C** Low level of CerS4, and therefore, C18-Cer prevents the inhibitory effect of histone deacetylase 1 (HDAC1)/SP3 complex on human telomerase reverse transcriptase (hTERT) transcription leading to an activation of hTERT which has pro-proliferative effects

Ecker et al. investigated the lipidome of colorectal cancer tissue in three patient cohorts and identified SM 32:1↑, SM 34:1↑, SM 35:1↑, SM 36:1↓, SM 40:1↓, SM 42:2↓, SM 42:3 and Cer d18: 1/18:0↓, Cer d18:1/20:0↓, Cer d18:1/18:1↓ and Cer d18:1/24:0↑ as lipids, which together could be used to clearly discriminate tumour from non-tumour colon tissue [25]. These data are a huge step forward in our understanding that lipids could also be used as biomarkers for colorectal cancer, but until now only a few laboratories perform lipidomics as a routine, so that lipidomic analysis is not a part of routine diagnostics. Other groups also found a decrease in Cer d18:1/18:0 and Cer d18:1/20:0 in colon tumour tissue compared to non-tumour tissue and an increase in the very-long-chain ceramide Cer d18:1/24:0 [42]. El-Hindi et al. found that CerS4, which is mainly responsible for the production of Cer d18:1/18:0 and Cer d18:1/20:0 in the colon, is decreased in colon cancer tissue compared to normal colon tissue. Mechanistically it was shown that in colon epithelial cells, CerS4 is downregulated following treatment of the cells with hypoxia, which may be one reason for the decrease in long-chain sphingolipids in colon cancer, considering that hypoxia is a common phenomenon in solid tumours due to improper vascularization [42]. How hypoxia leads to a decrease of CerS4 expression is not known, but several transcription factors that are predicted to bind at the CerS4 promotor such as the hypoxia inducible factor-1$\alpha$ (HIF1$\alpha$), activating protein-1 (AP-1), nuclear factor kappa-B (Nf$\kappa$B) and Sp1 are regulated by hypoxia (Fig. 3A) [20, 21, 102, 112]. The 3′-UTR of the CerS4 mRNA might also contribute to downregulation of CerS4 transcripts after hypoxia, as it contains a binding site for alcohol dehydrogenase 3′-UTR downregulation control element (ADH_DRE), a polyadenylation site, and a putative binding site for miR-339-5p (Fig. 3A). miR-339-5p has been shown to be downregulated after hypoxia–ischemia [115]. Until now, it is not known how these transcriptional and posttranscriptional regulatory elements are involved in CerS4 regulation after hypoxia. It has been shown that CerS4 is downregulated in K-Ras mutant tumours [38] and by depletion of Myc, which is a direct transcriptional regulator of CerS4 [62]. CerS4 is also downregulated by cancer therapeutics such as anastrozole and 5-fluoruracil in colon cancer cells [66]. Cell stress induces the cleavage of Myc, generating Myc-nick [17] suggesting that cell stress may be a mechanism leading to the downregulation of CerS4 in colon cancer cells. Which of these factors are involved in the downregulation of CerS4 in colon cancer tissue needs to be investigated in future experiments. Recently, it has been shown that CerS4 RNA can also undergo A-to-I (adenosine to inosine) RNA editing in pancreatic cancer. This RNA editing results in the loss of exon 3 and a truncated protein [65]. Whether RNA editing of CerS4 is also relevant in colorectal cancer remains to be shown, but RNA editing is also increased in colorectal cancer [90]. There are many possible mechanisms by which CerS4 can be downregulated in colorectal cancer; how this can influence tumour development is summarized in the next chapter.

To investigate the impact of CerS4 on tumour development and early carcinogenesis, we applied the AOM/DSS colon cancer model to CerS4 ko mice. CerS4 ko mice were highly sensitive to AOM treatment, as they show a severe disease score, defined by body weight, stool consistency, bleeding and posture, during the AOM/DSS treatment period in comparison to wt mice. But at least they showed no more tumour burden compared to wt mice. Further cell-type-specific depletion of CerS4 indicated that an intestinal epithelial-specific CerS4 depletion in mice has a protective effect against the AOM/DSS-induced colon carcinogenesis, suggesting that loss of CerS4 in colon epithelial cells protects cells for tumour-promoting signals [26]. These data are consistent with human in vitro data, showing that in non-aggressively growing colon tumour cells, depletion of CerS4 inhibits cell proliferation. Instead, the Human Protein Atlas data show that reduced CerS4 expression in late-stage colon tumours is associated with a worse outcome in these patients [42]. Accordingly, in in vitro experiments, downregulation of CerS4 in aggressively growing tumour cells such as SW620 and HCT-116 has been shown to have pro-proliferative effects in a 3D organoid model in vitro and in a xenograft tumour model in nude mice. The pro-proliferative effect of CerS4 downregulation in these cells was accompanied by metabolic changes such as an increase in metabolites of the pentose phosphate pathway that deliver building blocks for DNA and RNA syntheses, an upregulation of various glucose- and monocarboxylate-transporter and a shift to a lower OCR (oxygen consumption rate)/ECAR (extracellular acidification rate) ratio, which indicates that these cells rely stronger on glycolysis [42]. Because of these changes in cell behaviour between non-aggressive and aggressive growing colon tumours, we believe that proteins that are responsible for tumour development and for tumour progression are affected by CerS4 generated sphingolipids. One of these proteins might be the inhibitor 2 of protein phosphatase 2A (I2PP2A). C18:0 Cer has been shown to bind directly to a region localized in helix 7 of I2PP2A, decreasing the interaction between I2PP2A and protein phosphatase 2A (PP2A) which leads to an activation of protein PP2A [68]. Interestingly, when I2PP2A is decreased in cells, the interaction with C18-Cer leads to a degradation of the oncogene c-Myc by PP2A, but when I2PP2A is overexpressed, the interaction with C18-Cer has a more pro-proliferative effect (Fig. 3B) [68]. PP2A functions as a tumour suppressor in colon cancer, so its interaction with I2PP2A and C18-Cer or other sphingolipids may be differentially affected at early and late stages of colon cancer development, leading to opposite effects [19]. Furthermore, C18-Cer has been shown to interfere with the SP3/ histone deacetylase 1 (HDAC1) complex, resulting in SP3 deacetylation and repression of the human telomerase reverse transcriptase (hTERT) promoter [107, 109, 110]. This interaction is specific to C18-Cer and could not be mimicked by C16-Cer [110]. hTERT is a driver of oncogenesis, increasing proliferation, survival and anti-apoptotic signals. Therefore, low level of CerS4 and C18-Cer counteracts the inhibitory effect of HDAC1/SP3 on the hTERT promoter, increases hTERT transcription and has pro-proliferative effects at least in aggressively growing tumours (Fig. 3C).

In conclusion, the reduction of the long-chain ceramides Cer d18:1/18:0 and Cer d18:1/20:0 is a critical point in colorectal cancer development, occurs at an early stage of colorectal cancer and has pro- and anti-proliferative effects, depending on the different genes deregulated simultaneously. However, low expression level of CerS4 in late-stage colorectal tumours is associated with a worse prognosis in these patients.

## CerS2 in colon cancer development

**Fig. 4:**
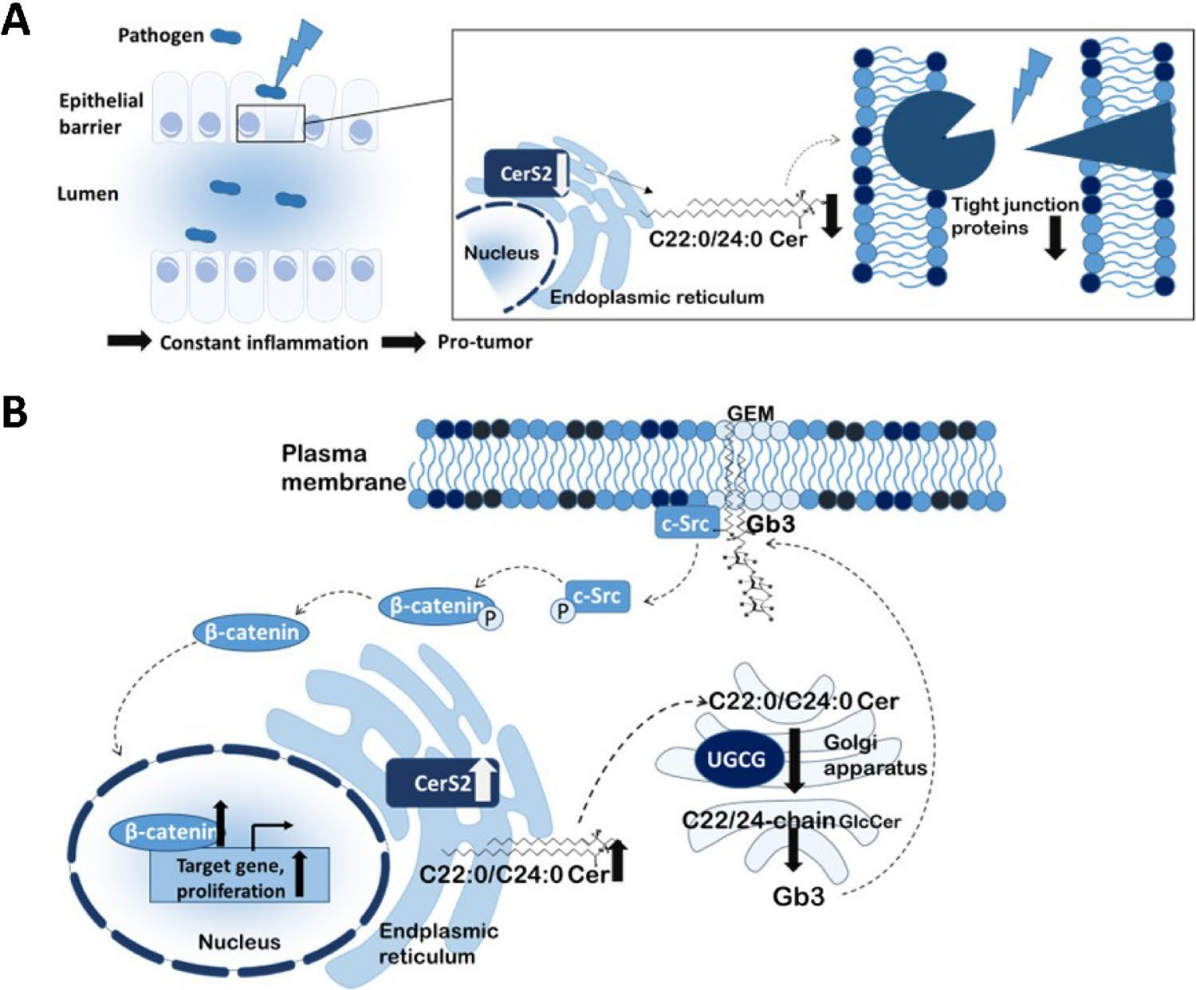
Influence of imbalanced CerS2 expression on colon tumour development. **A** Knockdown of CerS2 in colon epithelial cells leads to a decrease of very long chain Cer (C22/24). Changes in the lipid status at the plasma membrane inhibits tight junction formation and impairs barrier integrity. Pathogens can migrate through the leaky epithelial barrier and cause constant inflammation. Constant inflammation in colon tissue triggers colon tumour development. **B** Overexpression of CerS2 leads to an increase of very-long-chain Cer (C22/24) which are preferentially used by the UDP-glucose ceramide glucosyltransferase (UGCG) to generate GlcCer that are further metabolized to globotriaosylceramide (Gb3). Gb3 interacts together with c-Src in glycolipid-enriched membranes (GEMs) and leads to phosphorylation of c-Src which subsequently dephosphorylates β-catenin. De-phosphorylated β-catenin translocates into the nucleus and acts as a transcription factor on multiple target genes that promote proliferation and therefore tumour formation

Very-long-chain ceramides (Cer d18:1/22:0, Cer d18:1/24:0 or Cer d18:1/24:1) which are mainly produced by CerS2 increase in colon cancer tissue in comparison to normal colon tissue [14, 42, 45]. CerS2 expression is regulated transcriptionally, post-transcriptionally and post-translationally [9, 57, 102, 103]. In human colon cancer tissue, CerS2 expression increases with tumour stage [42]. The impact of CerS2 on colon carcinogenesis was studied by two groups applying the DSS- and AOM/DSS-induced colitis colon cancer model to CerS2 knockout mice [50, 71]. In their study, CerS2-deficient mice were much more sensitive against DSS-induced colitis and developed more colon tumours after AOM/DSS treatment. The enhanced sensitivity was related to a disruption of the colon epithelial barrier function demonstrated by loss of zonula occludens-1 (Zo-1) and occludin in colon epithelial cells of CerS2-ko mice as well as in CerS2-depleted Caco-2 cells (Fig. 4A) [71]. The increased tumour formation in CerS2-ko mice is probably related to the loss of barrier function which aggravates inflammatory reaction in the colon due to the infiltration of immune cells into the colonic tissue. Although the AOM/DSS model is a common model to study colon cancer development in vivo and has its justification in the imitation of inflammation-dependent tumour development, it is not the best model to imitate spontaneous colon cancer development, which is mainly based on spontaneous occurring mutations in oncogenes or tumour-suppressor genes. In colorectal cancer mutations in the APC gene leading to loss of function of the protein is one of the first events [52]. In mice organoids, the loss of APC leads to a strong increase in very-long-chain ceramides such as Cer(d18:0/22:0), Cer(d18:1/24:1) and hexyl-ceramides HexCer(d18:1/22:0) that are produced by CerS2 [48]. Interestingly, deficiency of SM in the plasma membrane is associated with an increased release of arachidonic acid (AA) induced by the secretory phospholipase A2 (sPLA2) [69]. Activation of sPLA2 might be related to a direct competition between SM and oxidized palmitoyl arachidonyl phosphatidylcholine (oxPC) for the active site of sPLA2 [53, 91]. AA is a precursor of many active lipids like prostaglandins, leukotrienes and epoxyeicosatrienoic acids that have been shown to be involved in colorectal cancer progression [13]. Therefore, the loss of the APC function is probably not sufficient to trigger colon cancer, but downstream signalling pathways that can be induced by sphingolipid alterations probably enhance the tumour-promoting effect [79]. How much CerS2 is involved in this effect is not entirely clear and has to be investigated in future experiments. Overexpression of CerS2 and co-expression with CerS6 or CerS4 in colon cancer cells have been shown to promote proliferation, whereas overexpression of CerS4 and CerS6 had rather anti-proliferative effects [36, 37]. Very-long-chain ceramides are preferentially converted to glucosylceramides by the action of UDP-glucose ceramide glucosyltransferase (UGCG) [111]. Inhibition of UGCG inhibits colon cancer cell growth in vitro and AOM/DSS-induced colon cancer development in mice and increases SM which is stored in multi-vesicular bodies [47]. GluCer are important for colon barrier maintenance [78] and can directly interact with the proto‐oncogene tyrosine‐protein kinase cSrc, thereby activating the $\beta$-catenin pathway (Fig. 4B) [84].These data indicate that very-long-chain GlcCer are important for cellular signalling pathways that drives colon cancer development.

In summary, an increase of CerS2, resulting in enhanced production of very-long-chain ceramides and GlcCer, has been detected in human colon cancer tissue, especially in late-stage tumours. Very-long-chain GlcCer interact with cSrc in glycosphingolipid-enriched microdomains and thus promote the development of colon cancer.

## CerS5 and CerS6 in colon cancer development

The lipidomic analysis of colon tumour tissue revealed that also C16-Cer, C16-HexCer and SM34 are elevated in tumour tissue in comparison to normal colon tissue [25, 42]. These long-chain ceramides are produced by CerS5 and CerS6. Both CerSs are expressed in colon tissue. Due to the high sequence similarity of both enzymes, it is hard to generate CerS5 or CerS6 specific antibodies that can be used to discriminate both enzymes by immunohistochemical staining. Depending on the used antibody, the literature data deviate in the expression level of CerS5 and CerS6 in colon tissue [30, 42]. As both enzymes are responsible for the production of long-chain ceramides, also the discrimination on sphingolipid level is not possible. Interestingly, both CerS5- and CerS6-ko mice are more sensitive to DSS-induced inflammation, but the molecular mechanisms are different. Loss of CerS5 or CerS6 has no effect on colon barrier function in untreated ko mice, but CerS6-ko mice show more inflammation after DSS treatment in comparison to wt mice which was related to an enhanced infiltration of neutrophils into the colon [39]. Instead, CerS5-ko mice were more susceptible to DSS-induced colitis and AOM/DSS-induced colon cancer which was related to disturbed T cell functions. CerS5 Vil1-Cre mice, where CerS5 is only depleted in colon epithelial cell, do not differ in their susceptibility against AOM/DSS-induced colon cancer in comparison to wt mice [27]. Both mice models show clearly that neither CerS5 nor CerS6 and therefore long-chain ceramide depletion impact colon epithelial cell barrier function.

In human colon cancer tissue, CerS6 expression rather increases, whereas CerS5 expression decreases especially in late-stage colon cancer [42]. The increase of CerS6 might be due to an enhanced expression of the long noncoding RNA (lncRNA) ceramide synthase 6 antisense RNA1 (CerS6 AS1), which leads to an enhanced stability of CerS6 mRNA and has tumour-promoting activities in colon cancer [4, 15]. In gastric or lung cancer cells, CerS6 has pro-tumourigenic effects that were related to the Jak-STAT signalling pathway and transcriptional upregulation of human telomerase reverse transcriptase at promoter level, respectively [98, 110]. However, overexpression or induction of CerS6 in human colon cancer cells has rather anti-proliferative and pro-apoptotic effects [36, 88, 97, 105]. This is also true for external addition of C16:0-Cer, by induction of lipotoxicity [80]. In this respect, it has been shown that C16-Cer produced by CerS6 binds to wt p53 and prohibits its interaction with MDM2 which rescues p53 from degradation by the proteasome pathway [29]. Interestingly, this is only observed after upregulation of C16-Cer by CerS6 but not by CerS5. These data indicate, that CerS5 and CerS6 are not interchangeable, and each enzyme has its own cellular value.

Depletion of CerS5 in aggressive growing colon cancer cells has pro-tumourigenic effects, whereas in non-aggressive growing colon cancer cells, it has rather anti-tumourigenic effects in *vitro* and in *vivo* [42]. These differences may be related to the different signalling pathways that are affected by CerS5 depletion in these cells. In aggressive growing colon cancer cells, CerS5 depletion leads to an upregulation of growth factor–related proteins like K-Ras and oncogenes like hepatoma-derived growth factor–related protein 2 (HDGFL2). In line with this, it has been shown that patients with low CerS5 expression in late-stage colon tumours have a poor prognosis [42].

In conclusion, CerS5 and CerS6 appear to have anti-proliferative effects in colorectal cancer. However, CerS6 is upregulated in colon tumour tissue, which may be related to the increased requirement of sphingolipids for membrane assembly in rapidly proliferating tumour cells. A decrease in CerS5 expression was only observed in metastatic colon tumours. Since both enzymes produce the same ceramide, the question arises as to why both enzymes are differentially affected in colon cancer tissue. We hypothesize that protein–protein interactions between CerS5 or CerS6 with other proteins or the location of these proteins in subcellular membranes may be responsible for the different effects on colon tumourigenesis, as the C16:0 ceramide produced by CerS5 and CerS6 affects different proteins [8]. But further studies are needed to prove this hypothesis.

## Outlook

**Fig. 5:**
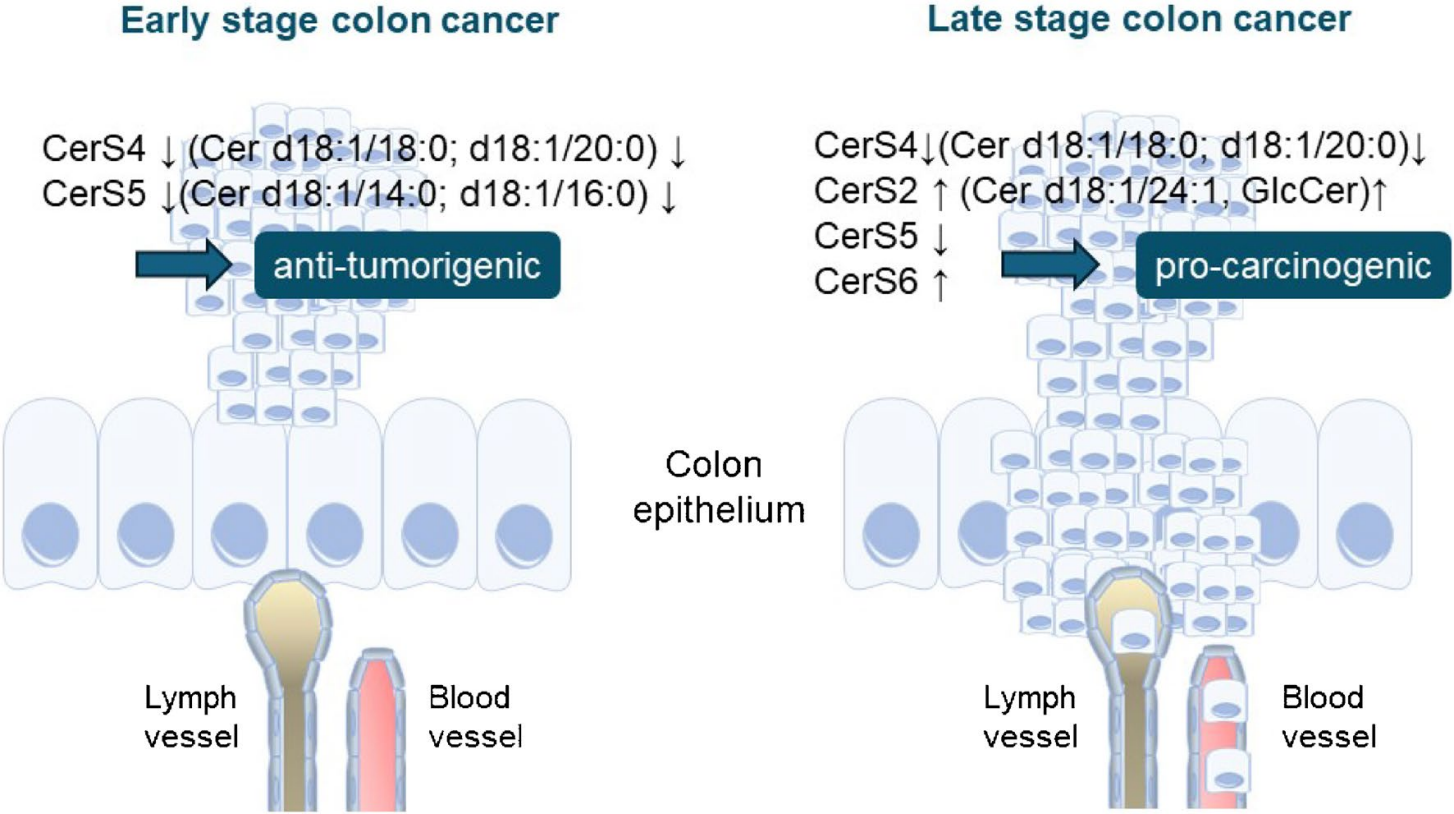
Schematic overview of how CerS and Cer of different chain lengths affect colon cancer at early and late stages

It has been shown that sphingolipids, especially sphingolipids of different chain lengths, are important for the physiological function of the colon and are deregulated in colon cancer (Fig. 5). However, we are still far from understanding the complex interactions between lipids and proteins. Further studies are needed to investigate the subcellular expression and functions of the different lipid species and their producing enzymes under physiological and pathological conditions. However, the accurate determination of sphingolipids in different cell types and tissues remains a challenge, as lipid measurements from tissue samples are often performed from tissue extracts. To gain more information on how sphingolipids influence the physiological functions of the colon, mass spectrometry imaging could be used to detect lipids, proteins, and metabolites in tissues. Mass spectrometric imaging using matrix-assisted laser desorption/ionization (MALDI) or desorption electrospray ionization (DESI) achieves a spatial resolution of < 10 µm [63], which is equivalent to single-cell resolution. To measure lipid-protein interactions, many techniques have been developed, each having advantages or disadvantages over the others [85]. According to our current knowledge, it is not sufficient to detect a protein-lipid interaction by static binding, because many lipid-protein interactions are very short-lived and often involve several proteins. Therefore, there are now a number of very sensitive methods that can also measure short-term interactions between proteins and lipids such as super-resolution microscopy or the use and detection of photoactivatable lipid probes [3]. The combination of spatial mass spectrometry imaging with highly sensitive methods for measuring short-term interactions can reveal how protein-lipid interactions work in the cell and which cellular mechanisms are affected by them.
